# Prospective study of human herpesvirus 8 oral shedding, viremia, and serological status among human immunodeficiency virus seropositive and seronegative individuals in Sao Paulo, Brazil

**DOI:** 10.1080/20002297.2017.1384287

**Published:** 2017-10-15

**Authors:** Paulo H. Braz-Silva, Tania R. Tozetto-mendoza, Laura M. Sumita, Wilton Freire, Michelle Palmieri, Alan M. do Canto, Vivian I. Avelino-Silva, Marina Gallottini, Philippe Mayaud, Claudio S. Pannuti

**Affiliations:** ^a^ Instituto de Medicina Tropical de Sao Paulo—IMTSP, Laboratorio de Virologia—LIM 52; ^b^ Faculdade de Odontologia—FOUSP, Departamento de Estomatologia, Disciplina de Patologia Geral; ^c^ Faculdade de Medicina—FMUSP, Departamento de Molestias Infecciosas e Parasitarias; ^d^ Departamento de Estomatologia, Disciplina de Patologia Oral e Maxilofacial;Universidade de Sao Paulo, Sao Paulo, Brazil; ^e^ Department of Clinical Research, Faculty of Infectious and Tropical Diseases, London School of Hygiene and Tropical Medicine, London, United Kingdom

**Keywords:** HHV-8, oral shedding, Kaposi sarcoma, HIV

## Abstract

Human herpesvirus 8 (HHV-8) is a gamma-herpesvirus and etiological agent of all forms of Kaposi sarcoma (KS). Saliva may play an important role in HHV-8 transmission in specific populations. Little is known about HHV-8 oral shedding pattern and the possible correlation with the HHV-8 serological profile and viremia. A prospective study was conducted of HHV-8 salivary excretion among human immunodeficiency virus HIV-seronegative (*n* = 47) and -seropositive (*n* = 44) homosexual men and HIV-seropositive women (*n* = 32) over a 6-month period with monthly HHV-8 serologies (immunofluorescence assays to identify antibodies to latent and lytic HHV-8 viral proteins, and a whole-virus HHV-8 enzyme-linked immunosorbent assay [ELISA]), monthly HHV-8 DNA serum/plasma detection, and daily self-collected oral rinses for HHV-8-DNA detection using real-time polymerase chain reaction. HHV-8 seropositivity was 51.1%, 63.6%, and 37.5%, in the three studied groups. There was no case of HHV-8 DNA detection in serum/plasma. Intermittent detection of oral HHV-8 DNA was observed during 5.1% (110/2,160) of visits among 28% (18/64) of HHV-8-seropositive individuals, all of whom were males and HHV-8 ELISA seropositive. In immunologically controlled populations of Brazil, HHV-8 oral shedding was limited to HHV-8-seropositive men, occurred infrequently and intermittently, and was not linked to HHV-8 viremia, suggesting a limited potential for oral or blood transmission.

## Introduction

Transmission of human herpesvirus 8 (HHV-8), the agent of Kaposi sarcoma (KS) and other human immunodeficiency virus (HIV)-associated rare conditions, has been the subject of conflicting evidence [1]. Oral samples have been consistently shown to have the highest rates of HHV-8 viral detection compared to semen, urine, urethral, vaginal, and anal samples, suggesting that saliva may play an important role in HHV-8 transmission [2–9]. On the other hand, transmission through blood and blood transfusion remains a controversial issue with no conclusive evidence [10–12]. Studies among blood donors outside KS endemic areas have shown very rare detection of HHV-8 DNA in blood samples, even among HHV-8-seropositive individuals [10,11].

Associations between HHV-8 serological status and HHV-8 DNA oral shedding are also not clear, with some studies even showing HHV-8 oral shedding in HHV-8-seronegative individuals [13]. The lack of a gold standard for HHV-8 serological status further compounds the limited ability to determine ‘true infection’ status [14].

This study aimed to assess prospectively the frequency of HHV-8 detection in saliva, serum, and plasma and their association with HHV-8 serological status among HIV-seronegative and -seropositive men who have sex with men (MSM) and HIV-seropositive women—groups that have a high probability of being infected with HHV-8.

## Methods

### Study participants and sample collection

A convenience sample of individuals were included from three groups: HIV-seronegative MSM, HIV-seropositive MSM, and HIV-seropositive women. Volunteer patients either self-presenting or referred by the STD Training Center/AIDS-SP in Sao Paulo for dental care were consecutively recruited at the Dental Special Care Center of the School of Dentistry, University of Sao Paulo, Brazil, where the purpose and procedures of the study were explained. The patients had no history of KS or other lesions associated with HHV-8. All patients signing informed consent were invited to a prospective follow-up over a 6-month period consisting of two sequential 3-month periods separated by 60 days, with biweekly appointments at the dental clinic where patients would bring their daily (Monday to Friday) self-collected mouthwashes. Self-collection commercial oral rinse kits (Listerine®, Johnson & Johnson, Sao Paulo, Brazil) were distributed to each patient, consisting of 50 mL plastic tubes (pre-labeled for the collection day) and a bottle of Listerine®. All participants were instructed to use 5 mL of the moutwash solution for 1 min at night and to collect all the contents of the oral rinse in the tube. Samples were stored in patients’ homes in a refrigerator at 4°C until the next dental appointment. The aim was to collect a total of 120 oral rinses from each patient. If a patient had not submitted this number, the collection period was extended up to 4 weeks (2 weeks in each period of 3 months) to attain that number. In addition, blood samples were drawn at the study entry and at monthly appointments. All samples were stored at −80°C until testing.

### Serological assays

Six serum samples from each patient were tested without knowledge of other results in a random patient and samples order for HHV-8 serologies at the Laboratory of Virology, Institute of Tropical Medicine, University of Sao Paulo, using in-house immunofluorescence assays (IFA) to identify antibodies to latent and lytic HHV-8 viral proteins, and a whole-virus HHV-8 enzyme-linked immunosorbent assay (ELISA) for immunoglobulin G detection, as described previously [14].

### HHV-8 molecular assays

#### DNA extraction

DNA was extracted from oral rinse, serum, and plasma samples (200 μL) using a NucliSENS® easyMAG® (bioMerieux, Durham, NC), an IVD-labeled automated system for total nucleic acid extraction based on magnetic silica technology, according to the manufacturer’s instructions. DNA concentration was mesured by spectrophotometry at 260 nM in a NanoDrop™ (Thermo Fisher Scientific, Waltham, MA). Beta-actin DNA was also detected in all samples by using real-time polymerase chain reaction (PCR), according to the manufacturer’s protocol (Taq Man DNA Template Reagents Kit; Applied Biosystems, Foster City, CA), in order to evaluate the viability of the DNA isolated from each sample.

#### ORF 26 real-time PCR.

Oral rinses, serum, and plasma samples were analysed in triplicate for HHV-8 DNA detection and quantification using real-time PCR, as described [11]. Quantities of HHV-8 DNA were log_10_ transformed to normalize values for analysis. Samples with ≥5 copies/mL (0.7 log_10_) were considered positive.

### Statistical analyses

The proportions of patients and days with detectable HHV-8 DNA in oral rinse, serum, and plasma samples were compared across the groups and by serological assay using chi-square statistics. For the quantitative analysis of viral shedding, the median values of HHV-8 DNA were compared among samples with detectable HHV-8 using the Kruskal–Wallis and Wilcoxon rank-sum tests. Inter-assay (serology) concordance (overall, positive, and negative agreements) was calculated using simple proportions. Performance of serological assays to detect HHV-8 shedding was calculated using standard 2 × 2 tables, and results are given with their 95% confidence intervals (CI). Data were analysed using Stata v14 (Stata Corp., College Station, TX).

## Results

Overall, 123 individuals (47 HIV-seronegative MSM, 44 HIV-seropositive MSM, and 32 HIV-seropositive women) were enrolled in this study from March to May 2013, and they were followed up for a 6-month period. Participants’ characteristics are shown in Table 1. All HIV-seropositive individuals were taking highly active antiretroviral therapy (HAART), and around 80% had undetectable HIV plasma viral loads (threshold for detection: 50 copies/mL) and high CD4 + T-lymphocyte counts (68.1% of men and 75.0% of women with CD4+ count ≥500 cells/mm^3^) at enrolment.

**Table 1.** Participants’ characteristics and HHV-8 antibody and DNA detection among HIV-positive and HIV-negative patients in Sao Paulo, Brazil.

	Male HIV negative, *N* = 47	Male HIV positive, *N* = 44	Female HIV positive, *N* = 32	*p*-Value
*Characteristics*				
Age (years), median (interquartile range)	30 (26–37)	40 (35–45)	42 (39–52)	<0.01^a^
Proportion with CD4+ >500 cells/mm^3^ (%)	n/a	30 (68.1)	24 (75.0)	0.52
Proportion with undetectable HIV PVL (%)^b^	n/a	35 (79.5)	27 (84.3)	0.59
*HHV-8 serology positivity*				
ELISA (%)	18 (38.3)	24 (54.6)	6 (18.8)	<0.01^c^
IFA-LANA (%)	14 (29.8)	13 (29.6)	6 (18.8)	0.49
IFA-lytic (%)	8 (17.0)	10 (22.7)	4 (12.5)	0.51
Any assay (%)	24 (51.0)	28 (63.6)	12 (37.5)	0.08
*HHV-8 DNA detection among all patients*	N* = 47*	N* = 44*	N* = 32*	
Serum^d^	0	0	0	—
Plasma^d^	0	0	0	—
Saliva (per person analysis)				
Positive person/tested (%)	7/47 (14.9)	11/44 (25.0)	0	0.23^e^
Saliva (per sample analysis)^d^				
Positive sample/tested (%)	40/5,640 (0.7)	70/5,280 (1.3)	0	<0.01^e^
*HHV-8 DNA saliva detection among HHV-8-seropositive patients only*	N* = 24*	N* = 28*	N* = 12*	
Per person analysis				
Positive individual/tested (%)	7/24 (29.2)	11/28 (39.3)	0	0.71^e^
Per sample analysis^d^				
Positive sample/tested (%)	40/2,880 (1.4)	70/3,360 (2.1)	0	0.04^e^
Positive samples among shedders samples (%)	40/840 (4.8)	70/1,320 (5.3)	0	0.58^e^
HHV-8 DNA log_10_ copies/mL	2.8 (2.4–3.2)	3.7 (3.3–4.5)	0	<0.01^e^

Continuous variables are presented as medians and interquartile ranges; *P*-values are presented for comparison across three groups

^a^Male HIV-negative group was significantly different from male HIV-positive and female HIV-positive groups in pairwise comparison; difference between male HIV positive-and female HIV-positive groups was not significant.

^b^Threshold for detection 50 copies/mL.

^c^In pairwise comparison, ELISA positivity was significantly different between male HIV-positive and female HIV-positive groups only.

^d^Per sample analysis: six serum, six plasma, and 120 saliva samples per patient.

^e^Pairwise comparison for male HIV-negative and male HIV-positive groups.

HHV-8, human herpesvirus 8; HIV, human immunodeficiency virus; PVL, plasma viral load; ELISA, enzyme-linked immunosorbent assay; IFA, immunofluorescence assays.

At the end of the study period, all patients had produced 120 self-collected oral samples, six serum samples, and six plasma samples. Forty-two (34.1%) patients needed an extended period to collect the oral rinses (ranging from 3 to 20 samples for a range of 1 to 4 weeks).

Overall, 24/47 (51.1%) HIV-seronegative MSM, 28/44 (63.6%) HIV-seropositive MSM, and 12/32 (37.5%) HIV-seropositive women were positive by at least one HHV-8 serological assay over six time points (Table 1). Considering each assay separately, there was a higher positivity by ELISA (48/123; 39.0%), followed by IFA-LANA (33/123; 26.8%), and IFA-lytic (22/123; 17.9%).

The overall intra-assay concordance in 738 serum samples was 100% (738/738) for ELISA, 99.2% 732/738) for IFA-LANA, and 98.9% (730/738) for IFA-lytic, showing excellent reproducibility for all tests. Patients with discordant intra-assay results were arbitrarily considered positive because the majority (4/6) of their serum samples were positive. The intra-assay concordance for negative results was 100% for each assay (Table 2).

**Table 2.** Intra-assay concordance for six serum samples obtained from 123 patients patients in Sao Paulo, Brazil.

Assay	Overall concordance, % (*n*/*N*)	Concordance among positive results, % (*n*/*N*)	Concordance among negative results, % (n/N)	Kappa (95% CI) for overall	*p*-Value
ELISA	100% (738/738)	100% (288/288)	100% (450/450)	100%	<0.0001
IF-LANA	99.2% (732/738)	96.9% (192/198)	100% (540/540)	96%	<0.0001
IF-lytic	98.9% (730/738)	94.2% (130/138)	100% (600/600)	94%	<0.0001

HHV-8 DNA was not detected in any of the 738 serum or plasma samples after triplicate PCR testing. HHV-8 DNA was not detected in any oral samples of the 59 HHV-8-seronegative individuals (total 7,080 samples tested), but it was detected in 18/64 (28%) HHV-8-seropositive individuals: 7/24 (29.2%) HIV-seronegative MSM and 11/28 (39.3%) HIV-seropositive MSM (Table 1). The frequency of HHV-8 DNA detection in samples from shedders was 5.1% (110/2160) overall, 4.8% (40/840) in samples from HIV-seronegative MSM, and 5.3% (70/1320) in samples from HIV-seropositive MSM (*p* = 0.58), with a range of positive samples between 3/120 (2.5%) and 11/120 (9.2%) per patient. The median quantity of HHV-8 DNA detected among shedders was significantly higher among the HIV-seropositive compared to HIV-seronegative MSM (3.7 vs. 2.8 log_10_ copies/mL, *p* < 0.01; Table 1).

The HHV-8 serological profile and the performance of individual and combination serological assays to detect HHV-8 shedders among the 91 men is shown in Table 3. The evaluation did not include women, since none had any demonstrable shedding. The whole-virus ELISA had the highest sensitivity (100.0%; 95% CI 81.5–100.0), lowest specificity (67.1%; 95% CI 55.1–77.7), but high negative predictive value (NPV; 100.0%; 95% CI: 92.7–100.0). The best combination of assays was ELISA plus IFA-LANA for its high specificity (91.8%; 95% CI 83.0–96.9) without much loss of sensitivity (83.3%; 95% CI 58.6–96.4) and high positive predictive value (PPV; 71.4%; 95% CI 47.8–88.7; Table 3).

**Table 3.** Performance of serological assays for the detection of salivary HHV-8 DNA shedders among 91 men (47 HIV-seropositive and 44 HIV-seronegative MSM).

Serologic assay	Positivity, *n* (%)	Sensitivity % (95% CI)	Specificity % (95% CI)	Positive predictive value % (95% CI)	Negative predictive value % (95% CI)
ELISA	18 (100.0)	100.0 (81.5–100.0)	67.1 (55.1–77.7)	42.9 (27.7–59.0)	100.0 (92.7–100.0)
IFA LANA	15 (83.3)	83.3 (58.6–96.4)	83.6 (73.0–91.2)	55.6 (35.3–74.5)	95.3 (86.9–99.0)
IFA-lytic	6 (33.3)	33.3 (13.3–59.0)	83.6 (73.0–91.2)	33.3 (13.3–59.0)	83.6 (73.0–91.2)
ELISA + IFA-LANA	15 (83.3)	83.3 (58.6–96.4)	91.8 (83.0–96.9)	71.4 (47.8–88.7)	95.7 (88.0–99.1)
ELISA + IFA-lytic	6 (33.3)	33.3 (13.3–59.0)	93.2 (84.7–97.7)	54.5 (23.4–83.3)	85.0 (75.3–92.0)
ELISA + IFA-LANA + IFA-lytic	6 (33.3)	33.3 (13.3–59.0)	98.6 (92.6–100.0)	85.7 (42.1–99.6)	85.7 (76.4–92.4)
IFA-LANA + IFA-lytic	6 (33.3)	33.3 (13.3–59.0)	94.5 (86.6–98.5)	60.0 (26.2–87.8)	85.2 (75.6–92.1)

## Discussion

In these high-risk populations, 29% of HHV-8/HIV dually seropositive MSM, as well as 39% of HHV-8-seropositive/HIV-seronegative MSM, were shedding HHV-8 DNA at least once over a 6-month period, while none of the HHV-8/HIV dually seropositve women shed HHV-8. Among MSM, the frequency of oral shedding was low (around 5% of days), irrespective of HIV serostatus, despite the large number of samples (120) tested per individual. However, the median quantity of oral HHV-8 DNA during episodes was significantly higher among HIV-seropositive shedders. Compared to other serological assays, ELISA would be the best test to identify HHV-8 shedders, as it combines the highest NPV (no shedding detected among ELISA-seronegative individuals) and the highest sensitivity. The pattern of HHV-8 oral shedding was sporadic for all 18 shedding individuals, with shedding days ranging from 2.5% to 17% and very few shedding episodes occurring on consecutive days.

The present findings differ from other studies that have reported a high frequency (32–68%) of HHV-8 shedding and high viral loads among HIV-seropositive women in Africa [3,4]. These studies were carried out in KS endemic areas (Kenya) among very specific populations (sex workers who did not use HAART) [3,4]. The small number of HHV-8-seropositive women in the present study, the different participant characteristics, and the fact that Brazilian patients were immunologically well controlled on HAART may explain these different results. However, it should be considered that some studies on gender susceptibility to HHV-8 and KS report that women are more resistant than men to infection by HHV-8 and development of the disease, which suggests that female hormones may provide protection to women [15,16].

Few studies have systematically analyzed the frequency and dynamics of HHV-8 oral shedding in KS non-endemic areas [2,5–7,17,18]. In the United States, Pauk et al. analyzed 1,134 oropharyngeal samples obtained from daily oral collection among 23 HHV-8-seropositive MSM (including 14 HIV-seropositive men) for a period of approximately 50 days. They found oral HHV-8 DNA detected at least once for 13 participants (57%), with about 50% of those men having frequent shedding (defined as ≥35% positive samples) [2]. Another study from the same group showed frequent and intermittent HHV-8 oral detection: 44 MSM collected oropharyngeal samples for periods of between 25 and 135 days, and 27 (61%) showed at least one positive sample. However, the authors also described HHV-8 oral shedding as intermittent and sporadic [6]. A recent study compared HHV-8 oral shedding between diferent populations (United States, Peru, Cameroon, Uganda, and Kenya), and the authors did not find a regular pattern of HHV-8 oral shedding, with a great variability observed between participants [17].

The present study found a high prevalence of HHV-8 antibodies in all three groups, although this varied according to the assay used (ranging from 18.7% with IFA-lytic to 56.8% for ELISA). The lack of a gold standard for the serological diagnosis of HHV-8 remains an obstacle for comparing the sensitivity and specificity of each assay, making it difficult to establish what is the most reliable assay to discriminate true HHV-8 infected individuals from those who are not infected [14]. By testing six sequential serum samples blindly from each patient, the present study was able to show excellent intra-assay concordance for all three techniques. Taking into account all 738 samples from the 123 individuals participating in the study, the intra-assay concordance was ≥99% for all assays.

One objective of this study was to determine the performance of serological assays to identify HHV-8 oral shedders. The HHV-8 whole-virus ELISA assay had the highest sensitivity, confirming previous reports [9,11,14], a moderate specificity, and a high NPV. The combination with IFA-LANA assay increased specificity without losing much on sensitivity and had the highest PPV to detect shedders.

It was found that none of the 738 blood samples, including those from the 18 oral shedders, were positive for HHV-8 DNA, despite triplicate testing, confirming earlier findings that in KS non-endemic areas, HHV-8 detection in peripheral blood is very rare, even among HHV-8-seropositive individuals such as blood-bank donors [10,11]. Blood donation by MSM has raised concerns about the possibility of HHV-8 transmission, and in some settings, it has been proposed to impose a lifetime donation deferral for this group [12]. This recommendation would not be supported by the present findings, at least for HHV-8.

In conclusion, in immunologically controlled populations of Brazil, HHV-8 oral shedding was limited to HHV-8-seropositive men, occurred infrequently and intermittently, and was not linked to HHV-8 viremia, suggesting a limited potential for oral or blood transmission.
